# Structural Alterations from Multiple Displacement Amplification of a Human Genome Revealed by Mate-Pair Sequencing

**DOI:** 10.1371/journal.pone.0022250

**Published:** 2011-07-22

**Authors:** Xiang Jiao, Magnus Rosenlund, Sean D. Hooper, Christian Tellgren-Roth, Liqun He, Yutao Fu, Jonathan Mangion, Tobias Sjöblom

**Affiliations:** 1 Department of Immunology, Genetics and Pathology, Uppsala University, Uppsala, Sweden; 2 Applied Biosystems, Life Technologies, Stockholm, Sweden; 3 Applied Biosystems, Life Technologies, Beverly, Massachusetts, United States of America; 4 Applied Biosystems, Life Technologies, Renfrew, United Kingdom; Deutsches Krebsforschungszentrum, Germany

## Abstract

Comprehensive identification of the acquired mutations that cause common cancers will require genomic analyses of large sets of tumor samples. Typically, the tissue material available from tumor specimens is limited, which creates a demand for accurate template amplification. We therefore evaluated whether phi29-mediated whole genome amplification introduces false positive structural mutations by massive mate-pair sequencing of a normal human genome before and after such amplification. Multiple displacement amplification led to a decrease in clone coverage and an increase by two orders of magnitude in the prevalence of inversions, but did not increase the prevalence of translocations. While multiple strand displacement amplification may find uses in translocation analyses, it is likely that alternative amplification strategies need to be developed to meet the demands of cancer genomics.

## Introduction

Unbiased and scalable analyses of copy neutral mutations, such as translocations and inversions, have been enabled by sequencing technologies that determine paired ends from genomic DNA fragmented into defined sizes [1]. Further, sensitive detection of structural variants in complex genomes will benefit from paired-end sequencing of fragment libraries with large insert sizes [2]. One of the most interesting applications of paired-end sequencing is rearrangement detection in cancer genomes [3], [4]. As rearrangements in cancer genomes frequently involve repetitive sequences, the ability to span large regions in each mate-pair is crucial for breakpoint detection sensitivity. The construction of such large insert size libraries by current methods is inefficient, which is problematic since high quality tissue material from human cancers often is present in limiting quantities. For example, the current procedures for construction of mate-pair libraries with ∼3 kb inserts consume 30 µg of genomic DNA. Thus, there is a need for accurate and unbiased whole genome amplification (WGA) procedures in cancer genomics.

In the past decades, several approaches to perform whole genome amplifications have been developed, which can be categorized according to their working mechanisms into PCR-based amplification [5], [6], linker-adaptor based amplification [7], [8] and multiple displacement amplification (MDA) [9]. The application of PCR-based and linker-adaptor based amplification in cancer genome sequencing has been limited by the relatively short amplification length (usually <3 kb) and the error rate of 3*10^−5^. Therefore, MDA approach becomes commonly used for the purpose of sequencing, genotyping and comparative genomic hybridization (CGH) arrays (reviewed in [10]). Essentially, the highly processive phage phi29 DNA polymerase is added to template DNA along with random hexamer primers, which can yield up to 1000-fold amplification of the original DNA sequence. Such amplification is known to introduce false positive inversions when applied to prokaryotic genomes, and to introduce false positive nucleotide level mutations predominantly at nucleotide runs [11], [12], although the error rate of phi29 DNA polymerase is estimated to be less than 3*10^−6^ [13]. However, the spectrum and extent of structural genomic alterations introduced by MDA of mammalian genomes remains largely unknown. Further, it is unclear if the structural artefacts created by MDA can be filtered from true positive rearrangements without loss of sensitivity. We therefore sought to determine the effects on coverage, rearrangement detection sensitivity, and the prevalence of false positive structural alterations when MDA is used to amplify and mate-pair sequence a normal human genome.

## Materials and Methods

Two healthy volunteers donated blood samples for this study. This study was approved by the Regional Ethical Review Board of Uppsala (2007/116) and written consent was obtained from both participants.

Reference DNA was obtained by phenol-chlorophorm extraction of whole blood from a healthy female donor. Ten WGA reactions were carried out using the REPLI-g^®^ Mini kit (QIAGEN) according to the manufacturer's instructions. In each reaction, 10 ng genomic DNA were denatured, neutralized with REPLI-g denaturation buffer and neutralization buffer, respectively, and incubated in REPLI-g reaction buffer with phi29 DNA polymerase at 30°C for 16 hours. The DNA polymerase was inactivated by heating the samples for 3 min at 65°C, and the WGA reactions were pooled and extracted twice with phenol-chloroform followed by ethanol precipitation. Thirty µg of reference and WGA amplified DNA, respectively, were used to construct SOLiD2 mate-paired libraries. All steps were carried out in parallel to ensure identical reaction conditions for the reference and WGA amplified sample. Briefly, the DNA was sheared into fragments between 1.5 kb and 4.5 kb by HydroShear (Genomic Solutions) and end-repaired using End-It DNA end-repair kit (Epicenter Biotechnologies). Methylation of the EcoP15I sites in the samples was carried out using EcoP15I in the presence of S-adenosyl methionine followed by ligation of EcoP15I cap adaptors (5′-pACAGCAG-3′, 5′-CATGTCGTCp-3′) to both ends of the fragments. Next, the adapter ligated DNA samples were separated on a 0.8% agarose gel and DNA fragments ∼3 kb in length were recovered and purified. The sizes and concentrations of adapter ligated DNA strands were quantified using a Bioanalyzer kit (DNA 7500, Agilent). The samples were circularized using internal adaptors and digested with EcoP15I. Digested DNA was end-repaired using End-It DNA end-repair kit (Epicenter Biotechnologies) and ligated to P1 (5′-CCACTACGCCTCCGCTTTCCTCTCTATGGGCAGTCGGTGAT-3′, 5′-ATCACCGACTGCCCATAGAGAGGAAAGCGGAGGCGTAGTGGTT-3′) and P2 adaptors (5′-AGAGAATGAGGAACCCGGGGCAGTT-3′, 5′-CTGCCCCGGGTTCCTCATTCTCT-3′). The mate-paired libraries were captured and purified by streptavidin beads (Dynal M-280, Invitrogen) according to the manufacturer's instructions. The libraries were further nick-translated followed by PCR-based amplification. PCR products were separated on a 4% agarose gel and 150–160 bp library bands were recovered, purified, and verified using a Bioanalyzer kit (Agilent, DNA 1000). Throughout the library preparation procedure, DNA was purified and concentrated with QIAquick columns (QIAGEN) after each enzymatic reaction and PCR. Emulsion PCR was performed according to the manufacturer's manual (SOLiD2 System Templated Bead Preparation Guide, Applied Biosystems) before SOLiD sequencing. Subsequently, 25 nt mate-pair sequences were collected on the AB SOLiD2 instrument.

Human genome sequences were downloaded from Ensembl (ftp://ftp.ensembl.org/pub/release-54/fasta/homo_sapiens/dna/, genome release NCBI36.54). Assembled autosomal chromosomes, chromosome X, and mitochondrial DNA were used as mapping reference. The SOLiD System Analysis Pipeline tool corona_lite (v4.0r2.0) was used for sequence mapping, mate-pairing, and SNP calling. Both tags were mapped to the genome separately by aligning 25 bp while allowing maximum 2 color space mismatches (corresponding to one SNP). Pairing identified whether the two tags were the expected distance apart in the genome or if there was a putative structural variation represented in the clone compared to the reference sequences. Second, for the tags that did not match within the insert size range, a mate-pair rescue step was performed. It is accomplished by using the hits to one tag as an anchor, and then scanning for the other tag in the region of the insert size range. The number of mismatches allowed in the other tag was limited by the total number of mismatches in both tags. Clone coverage was defined as the number of uniquely mapped read pairs multiplied by the clone insert size (the mode of the distribution of clone length detected by Bioanalyzer) and divided by the number of bases in the haploid human genome (3,022,646,526 bp).

Insertions and deletions were inferred from clone size using the AB Large InDel Tool (v1.0), which identifies deviations in clone insert size from a reference genome. Insertions and deletions up to 100 kb are inferred by identifying positions in the genome in which the pairing distance between mapped mate-pairs deviates significantly from what is expected at the given level of clone coverage. Inversions were detected by applying the AB Inversion tool (v1) to the mapped reads, which identifies reciprocal nearest neighboring start/end breakpoints to call full inversions [2]. Essentially, the number of mate-pairs supporting a breakpoint is counted for each base pair, and if the number of such mate-pairs exceeds a threshold the base pairs with the local maximum constitute the candidate breakpoint range. If two neighboring breakpoints A and B are identified, but later another breakpoint C is found which is closer to A than B, then A and C becomes a new pair and B becomes an ‘orphan’ breakpoint. The inversion score is defined as the harmonic mean of the number of mate-pairs supporting the left and right breakpoints of the inversion. The orphan breakpoints are paired by default with their nearest neighbor if they produce higher inversion scores than the corresponding normal inversions. The inversions observed in the non-amplified DNA sample that were supported by at least four mate-pairs spanning each breakpoint and had both putative breakpoint ranges known within 2 kb were selected for further validation.

Translocations were detected by first extracting all mate-pairs where the two tags mapped on different chromosomes and sorting these mate-pairs by the reverse tag, then separating q-q from p-q translocations, grouping mate-pairs that had both forward and reverse tags mapped within 3.2 kb of each other, and applying a cut-off requiring at least 2 mate-pairs to score a translocation. To remove artefactual translocations arising at the boundaries of uncharted regions of the genome, we removed all translocations where one of the tags in the mate-pair mapped within 5 kb of 25 or more consecutive N:s. By comparing to translocations identified in another normal control genome, false mapping “hotspots” were defined as areas containing significantly (at 99.9% confidence) higher numbers of reads than the genomic average in the unamplified control set. All translocations with tags mapped in those hotspots were considered as false positives and removed. Translocations detected to be recurrent in another normal genome by the same method were excluded to get rid of false positives caused by incomplete reference genome sequence. The translocations remaining in either the non-amplified or the MDA sample after application of these filters were chosen for further validation by PCR. All primer pairs to amplify across the breakpoint sequences were generated using Primer3 (http://foller.wi.mit.edu/primer3/input.htm) with the predicted breakpoint range in the samples as template. Next, the primer pairs were filtered by In-Silico PCR (http://genome.ucsc.edu/ispcr) to avoid false positive products (Table S1). After PCR amplification and gel-purification, the products were sequenced with forward and reverse primers by Sanger sequencing. The obtained sequences were aligned against human genome release NCBI36/hg18 using BLAT (http://genome.ucsc.edu/cgi-bin/hgBlat) to determine breakpoint positions.

## Results and Discussion

To determine the effects of MDA under conditions suitable to rearrangement discovery in cancer genomes, we generated large insert mate-pair libraries from the native and WGA genome of a healthy female donor. After amplification, the DNA was sheared to a suitable length for rearrangement analyses (∼3 kb). Mate-pair libraries were constructed and 25 nt sequences from each end of the mate-pairs were collected using the SOLiD2 instrument (Applied Biosystems). The integrity of the mate-pair library preparations was confirmed by correlation analysis of the chromosomal locations of the two tags in each pair (Figure S1). As both ends of each insert are supposed to be sequenced at equal efficiency, one would expect an equal amount of 5′ tags and 3′ tags in each genomic region (Pearson r = 1). The excellent correlation between the locations of the two reads in each pair demonstrates the integrity of SOLiD mate-pair library preparations, as artefacts such as chimeric clones arising during library construction would decrease these correlations.

**Figure 1:**
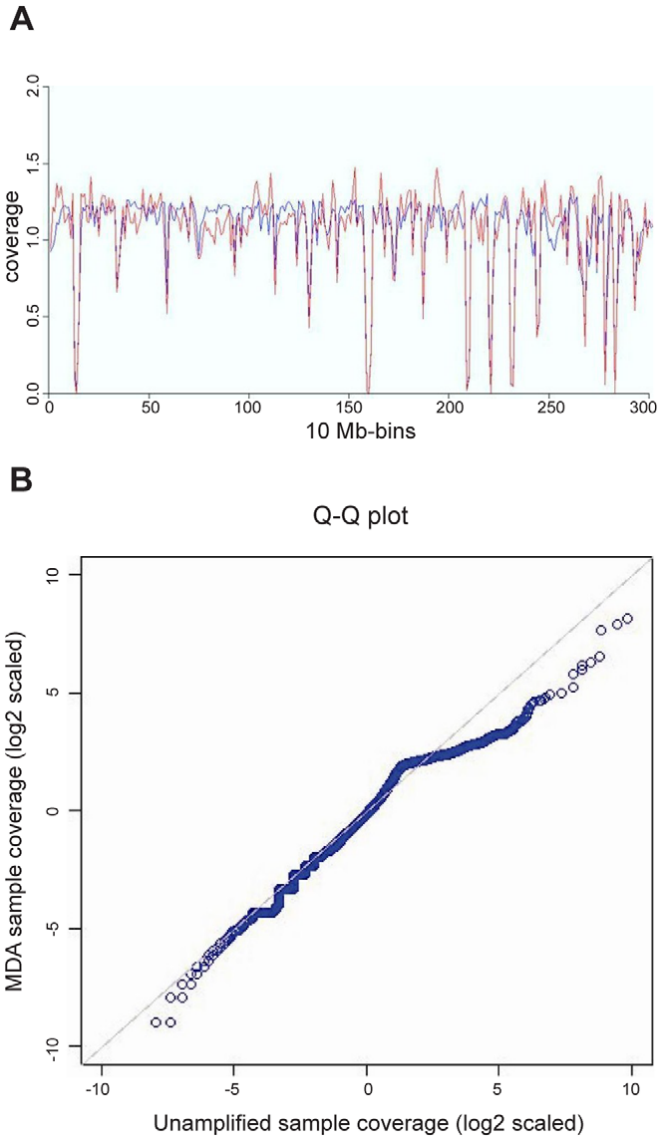
Introduction of coverage bias by multiple displacement amplification. A. Fold base coverage per bin of 10 MBp consecutive bases before (blue line) and after (red line) whole genome amplification. B. Logarithmic quantile-quantile plot of the binned distributions (bin size 1000 nt) of the sum of the coverage of both tags of a mate-pair.

A summary of the mate-pair sequencing data collected from the control and MDA amplified genome is presented in Table 1. We first compared the read coverage before and after MDA (Figure 1). The local coverage variation is greater in the MDA sample than in the non-amplified sample. Bins with average fold base coverage >4 are more abundant in the non-amplified sample than in the MDA sample as seen in Figure 1B where the points are below the x = y line for x>2. The low contig coverage correlation (Pearson r = 0.65) between non-amplified and amplified DNA also implies that MDA biases sequence representation in the end product. For comparison, the correlation between replicate mate-pair sequencing of the same sample is expected to exceed 0.99 (Applied Biosystems, unpublished observation).

**Table 1:** Analysis of structural alterations in a human genome before and after MDA.

	Control	MDA
**Clone insert length (nt)**	3199	3274
**Sequence coverage**	1.07	0.94
**Clone coverage**	65.1	53.8
**Non-redundant mate-pairs mapped**	64.6×10^6^	57.1×10^6^
*normal*	95.6%	87.4%
*spanning putative deletions*	0.09%	0.14%
*spanning putative insertions*	0.03%	0.09%
*spanning putative inversions*	0.12%	6.2%
*spanning putative tandem repeats or double inversions*	0.06%	0.5%
*spanning putative translocations*	4.1%	5.7%
**Insertions (size range 124–2354 bp)^¤^**	1019	416
**Deletions (size range 142–95772 bp)^¤^**	2879	2418
**Inversions^#^**	31	7071
**Translocations^#^**	424	105
*near uncharted reference§*	136	14
*in false positive mapping hotspots*	251	77
*recurring in another normal genome*	20	9
*validated by PCR*	7	3

¤, insertions and deletions were detected with the AB Large InDel tool. #, breakpoint(s) supported by at least 4 mate-pairs. §, translocations reported where one tag maps within 5 kb of 25 or more consecutive N:s in the reference genome were removed.

**Figure 2:**
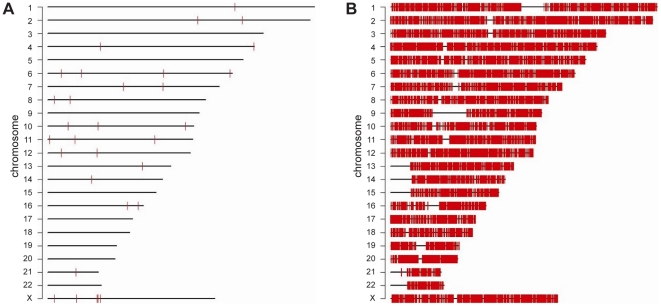
Multiple strand displacement of a normal human genome introduces inversions. Inversions (red bars) supported by at least 4 independent mate-pairs after shotgun genome sequencing of a non-amplified (A) and MDA amplified (B) genome.

**Figure 3:**
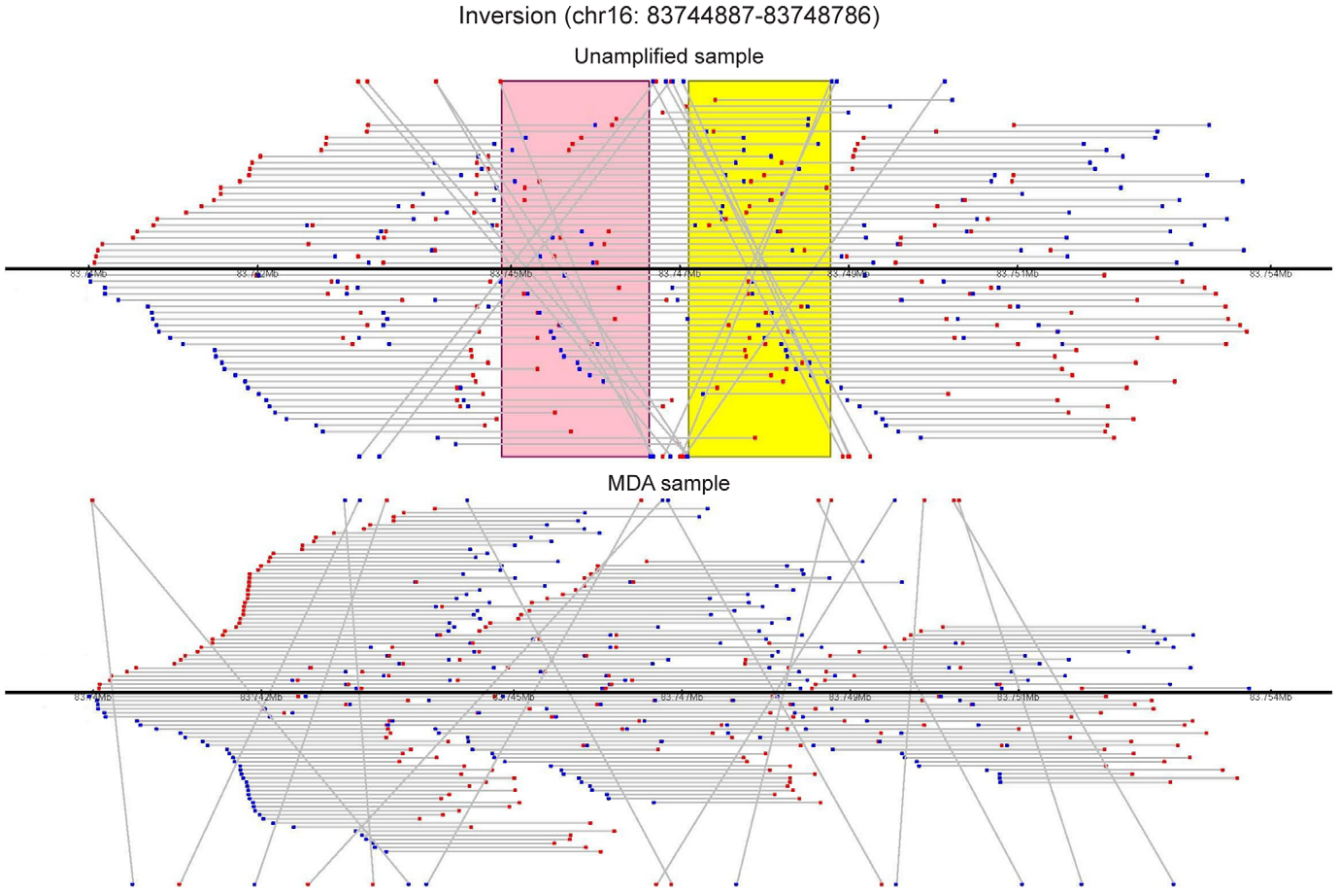
Loss of sensitivity in breakpoint detection by multiple strand displacement-induced inversions. Forward (blue) and reverse (red) tags in mate-pairs (grey line) surrounding the start and end breakpoints of the inversion chr16∶83744887–83748786 along with 5 kb flanking regions on chromosome 16 (black line). The tags mapping to the forward chromosome strand are plotted above the chromosome line, and the tags mapping to the reverse strand are plotted below. The inversion start and end regions identified are shown as pink and yellow bands, respectively.

Next, mate-pairs were mapped and it was noted that 4.4% (control) versus 12.6% (MDA) of mate-pairs were not expected based on the reference genome, indicating that MDA may induce a large number of structural alterations. Whereas the increases in mate-pairs supporting deletions, amplifications, and translocations in the MDA sample were within one order of magnitude, MDA caused a ∼50-fold increase in non-redundant mate-pairs spanning putative inversions and a ∼10-fold increase in such tags spanning double inversions. If more stringent criteria were applied by requiring four independent mate-pairs supporting each breakpoint to call an inversion, the MDA sample had >200-fold more inversions evenly distributed across the genome (Figure 2). Thirty-one putative inversions observed in the non-amplified DNA sample were supported by four or more mate-pairs, and 20 of these had both breakpoints known within 2 kb. The latter inversions were chosen for validation by PCR and Sanger sequencing. Eight of 20 inversions tested yielded PCR products from both breakpoints, 8 from one breakpoint, and 4 did not yield any PCR product. Of the 16 inversions that were supported by PCR products, Sanger sequence was obtained across one or two breakpoints in 12 inversions and these were considered true positive inversions (Figure S2). The median sizes and distributions of inversions in the non-amplified (median 5995, range 1764–54033864 bp) and amplified (median 72604, range 2216–3681636 bp) samples were different (p = 3.9 * 10^−9^, Wilcoxon test) (Figure S3). However, we were not able to formulate simple criteria to discriminate false positive inversions from true positive inversions based on inversion size alone. This may in part be due to the small number of inversions in the non-amplified sample. The clone coverage per sample of the present study is ∼5-fold less than that of [2], where 91 inversions were observed in NA18507 by combined SOLiD mate-pair and fragment sequencing, which may explain why we observed only 31 inversions in the control genome with each breakpoint supported by 4 or more mate-pairs. All of the inversions in the normal sample whose breakpoints could be amplified and/or sequenced represent previously known variants [14], which supports the ability of the analysis pipeline to detect true inversions. When allowing a minimum of 50% inversion overlap, and requiring 4 mate-pairs to support each inversion breakpoint, only 4 of the 12 confirmed inversions were detected in the MDA sample. Further relaxing these requirements to 25% overlap, and including orphan breakpoints, increased this number to 5 inversions. While the low degree of overlap between validated inversions in the control and MDA sample may be partly explained by insufficient coverage, it is more likely that spurious false positive inversions in the MDA sample mask the true positive inversions resulting in lower detection sensitivity given a similar level of global clone coverage (Figure 3). For comparison, the mate-pair data from regions of 10 true positive inversions is shown for the control and MDA genome (Figure S4).

As the genome sequence was derived from a healthy donor, the vast majority or all of the translocations detected are likely to stem from mapping errors in centromeric regions and sequences currently thought to be unique that are recurring in still uncharted regions of the human genome. Indeed, the numbers of translocations were reduced by removing mate-pairs where one of the two tags mapped within 5 kb of 25 or more consecutive ambiguous bases: 288/424, and 91/105 translocations remained in the unamplified and MDA samples, respectively. As a second step, 251 and 77 translocations mapped within false mapping hotspots were eliminated. Moreover, translocations detected to be recurrent in another normal genome were discarded. Following these *in silico* steps to remove likely false positives, seventeen and five putative interchromosomal translocations supported by at least 2 mate-pairs remained before and after MDA, respectively. Three translocations were observed in both samples. Eighteen out of a total of 19 different translocations were chosen for further validation by PCR using the non-amplified DNA sample as template. Nine of 18 translocations tested yielded PCR products, including 1 translocation observed in both samples and 6 detected only in the sample before MDA, which indicates existence of these translocations. Notably, the 2 translocations detected only in the MDA sample also yielded PCR products in the non-amplified DNA. We next performed PCR with the same primers in the genome of another healthy donor. The presence and sizes of PCR products were identical between the two genomes except for one primer pair which showed a complex band pattern in both samples (Figure S5). This indicates that most of the putative translocations detected in this genome are caused by mapping errors, and that phi29-mediated MDA does not induce false positive interchromosomal translocations.

Prior studies on MDA-induced inversions in bacterial genomes support a role for nearby displaced 3′ termini in initiating priming on nearby displaced 5′ termini [12]. The majority of chimeras observed after MDA of the *E. coli* K12 genome were inverted sequences with an intervening deletion with 80% of breakpoints stemming from within 10 kb of each other in the original sequence. However, inter-chromosomal translocations were not due to the simplicity of *E. coli* genome, which consist of only one circular chromosome. When amplification is performed on genomes with multiple chromosomes, the local character of the phenomenon observed by Lasken and coworkers implies that intra-chromosomal aberrant priming is more likely than mispriming involving different segments of DNA [12]. The increased prevalence of intrachromosomal aberrations, such as inversions and deletions, but not translocations in the MDA amplified human genome favors this hypothesis. Although the high false positive rate of intrachromosomal aberrations renders MDA inefficient in mapping such alterations, the low prevalence of false positive translocations may enable scientific or diagnostic uses for detection of inter-chromosomal rearrangements. However, the uneven sequence representation is likely to increase the false negative rate.

In conclusion, phi29-mediated whole genome amplification by multiple strand displacement introduces false positive structural aberrations, with an emphasis on inversions. As WGA entails a sequence representation bias and increases the subsequent structural mutation validation effort by >200-fold, its current incarnations have limited value in whole genome sequencing.

## Supporting Information

Figure S1 **Coverage correlation between mate-pair tags on chromosome 1.** Quantile-Quantile plot of the binned distributions of end tag coverage on a logarithmic scale before (A) and after (B) whole genome amplification. The apparent difference in the distributions that can be noted for low coverage is most likely an artefact of the representation (1000 bp bins) in addition to a greater sensitivity to random effects due to sparse data (Pearson r  = 0.95 and 0.87 respectively in A and B before taking logarithms).
(PDF)

Figure S2 **Identification of true inversions in a non-amplified genome by PCR-coupled Sanger sequencing.** Putative inversions identified by mate-pair sequencing of a normal human genome were validated by PCR amplification and sequencing. S, start point (breakpoint with lower genomic coordinate); E: end point (breakpoints with higher genomic coordinate).
(PDF)

Figure S3 **Different size distribution of MDA-induced inversions as compared to inversions in the human genome.** Box and whisker plot of inversion sizes in a genome before and after multiple strand displacement amplification.
(PDF)

Figure S4 **Loss of inversion detection sensitivity by spurious MDA-induced inversions.** Ten examples of mate-paired data from true positive inversions in normal (upper panels) or MDA (lower panels) DNA from a healthy individual. Forward (blue) and reverse (red) tags in mate-pairs (grey line) are surrounding the start and end breakpoints along with 5 kb flanking regions on the chromosome (black line). The tags mapping to the forward chromosome strand are plotted above the chromosome line, and the tags mapping to the reverse strand are plotted below. The inversion start and end regions identified are shown as pink and yellow bands, respectively.
(PDF)

Figure S5 **PCR validation of putative interchromosomal translocations detected in a non-amplified and MDA-amplified human genome.** Putative inversions identified by mate-pair sequencing of a normal human genome before and after MDA were validated by PCR amplification in non-amplified DNA of the same genome and another normal genome. The 2 approximate breakpoints of each translocation are listed. Genomic order as a negative number indicates that the translocation contains joints between a plus strand and a minus strand.
(PDF)

Table S1 **Primers used for PCR validation and sequencing.**
(PDF)
