# Supplementary figures and images for: Structural Alterations from Multiple Displacement Amplification of a Human Genome Revealed by Mate-Pair Sequencing
Source: PLoS One. 2011 Jul 22;6(7):e22250. doi: 10.1371/journal.pone.0022250 (PMC3142133; doi:10.1371/journal.pone.0022250)

**Figure S1**

A)

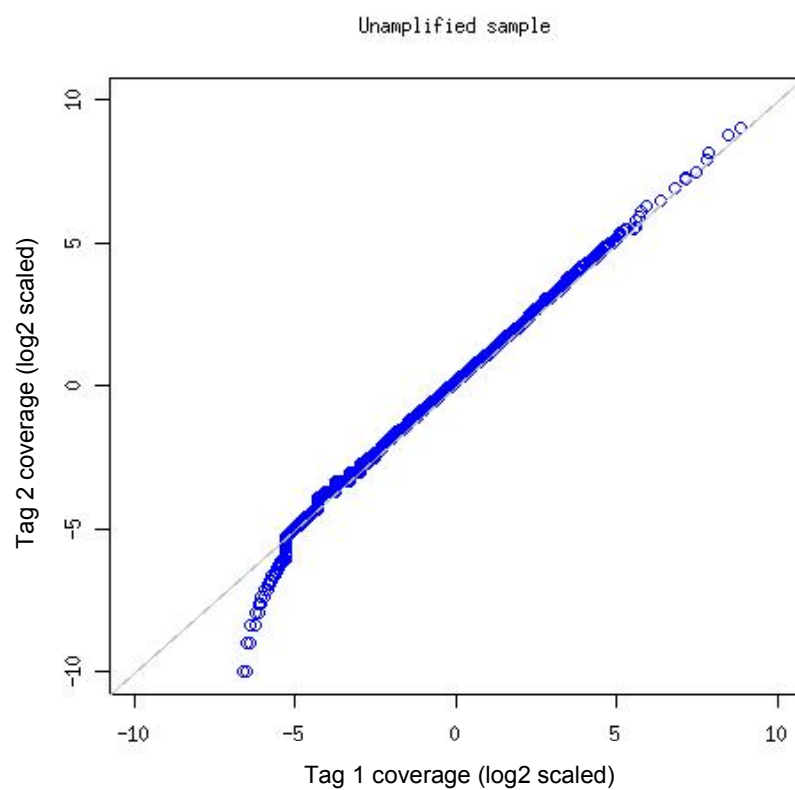

B)

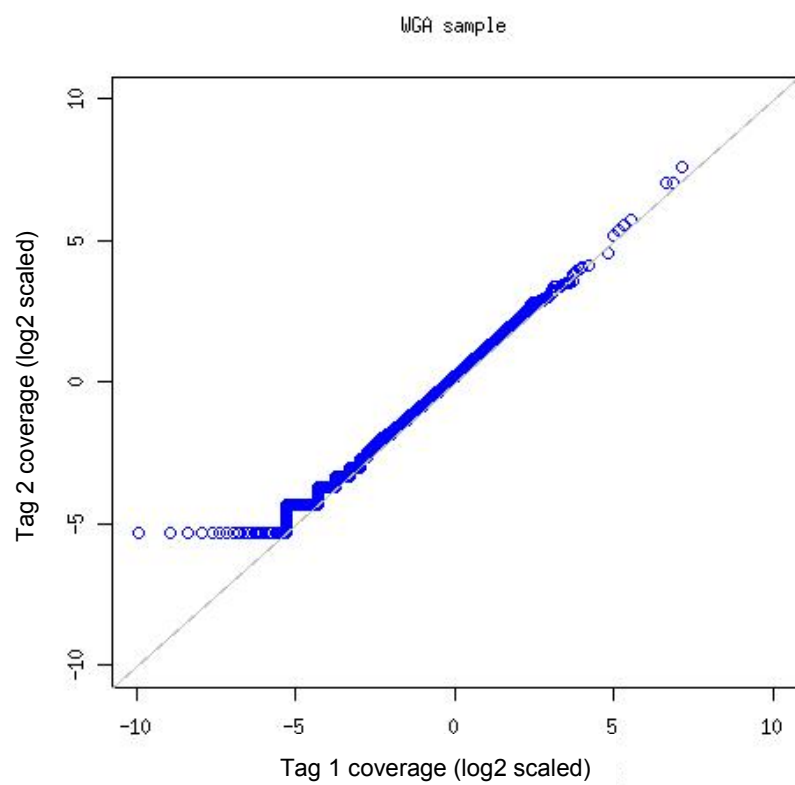

Supplement: Figure S1 — Coverage correlation between mate-pair tags on chromosome 1. Quantile-Quantile plot of the binned distributions of end tag coverage on a logarithmic scale before (A) and after (B) whole genome amplification. The apparent difference in the distributions that can be noted for low coverage is most likely an artefact of the representation (1000 bp bins) in addition to a greater sensitivity to random effects due to sparse data (Pearson r = 0.95 and 0.87 respectively in A and B before taking logarithms). (PDF) [file pone.0022250.s001.pdf]

Figure S2

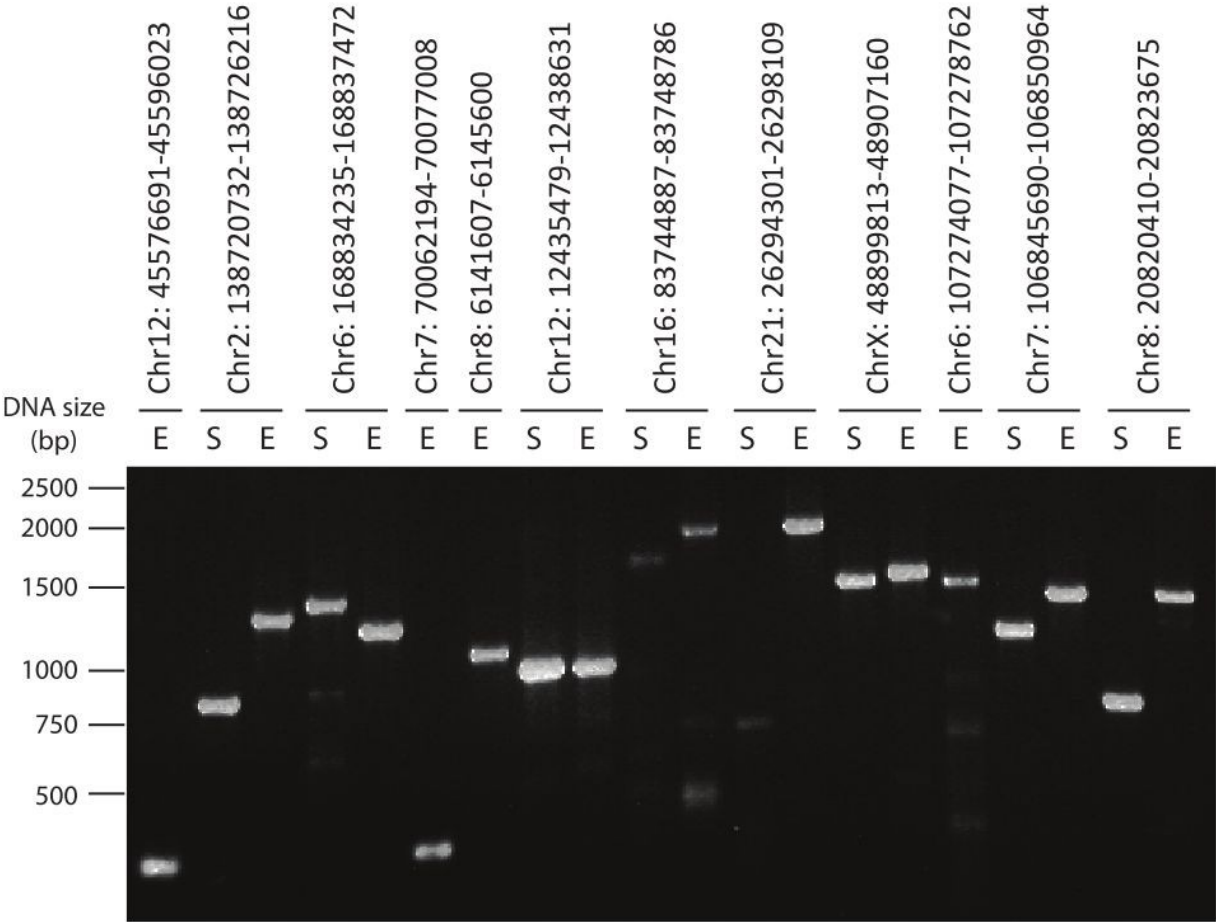

Supplement: Figure S2 — Identification of true inversions in a non-amplified genome by PCR-coupled Sanger sequencing. Putative inversions identified by mate-pair sequencing of a normal human genome were validated by PCR amplification and sequencing. S, start point (breakpoint with lower genomic coordinate); E: end point (breakpoints with higher genomic coordinate). (PDF) [file pone.0022250.s002.pdf]

**Figure S3.**

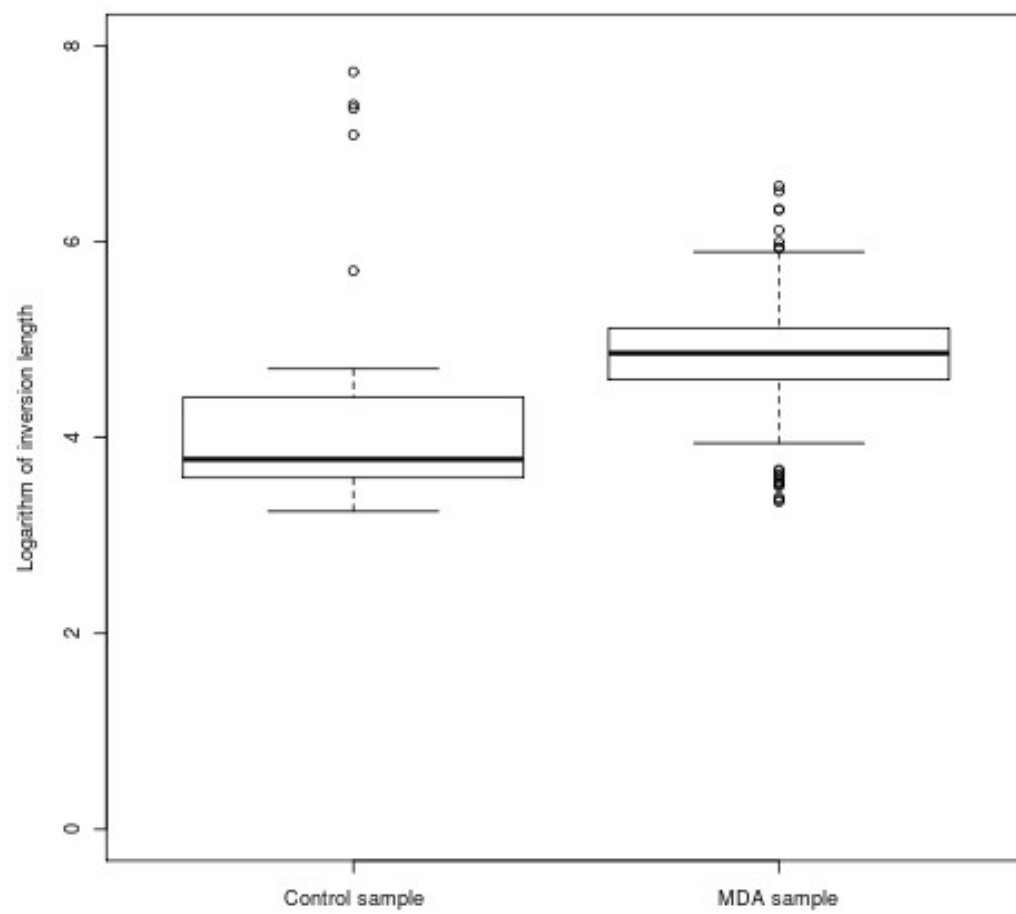

Supplement: Figure S3 — Different size distribution of MDA-induced inversions as compared to inversions in the human genome. Box and whisker plot of inversion sizes in a genome before and after multiple strand displacement amplification. (PDF) [file pone.0022250.s003.pdf]

Figure S5

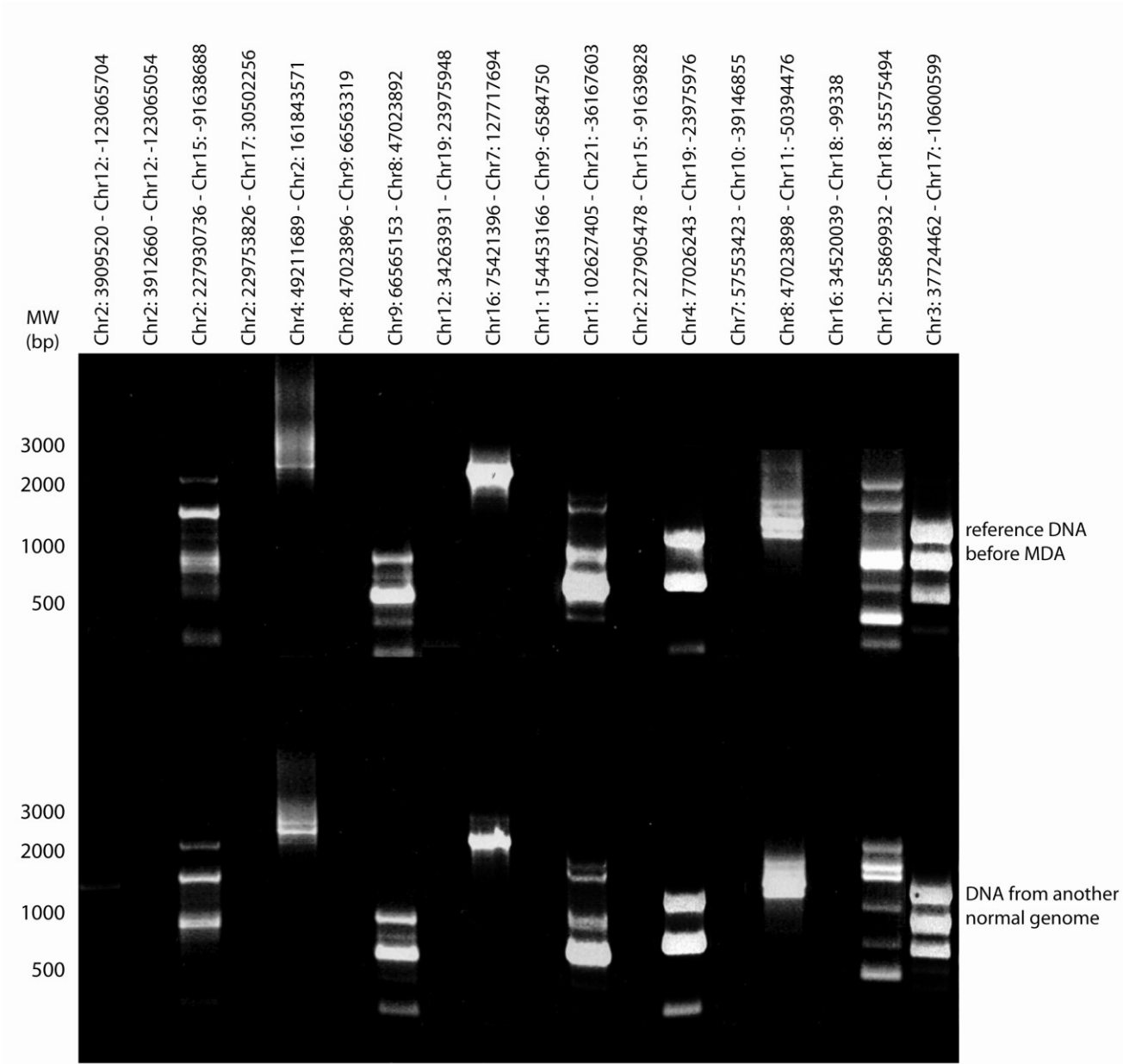

Supplement: Figure S5 — PCR validation of putative interchromosomal translocations detected in a non-amplified and MDA-amplified human genome. Putative inversions identified by mate-pair sequencing of a normal human genome before and after MDA were validated by PCR amplification in non-amplified DNA of the same genome and another normal genome. The 2 approximate breakpoints of each translocation are listed. Genomic order as a negative number indicates that the translocation contains joints between a plus strand and a minus strand. (PDF) [file pone.0022250.s005.pdf]
