# Supplementary material for: Structural Alterations from Multiple Displacement Amplification of a Human Genome Revealed by Mate-Pair Sequencing
Source: PLoS One. 2011 Jul 22;6(7):e22250. doi: 10.1371/journal.pone.0022250 (PMC3142133; doi:10.1371/journal.pone.0022250)

Figure S4

inversion (chr12:45576691−45596023)

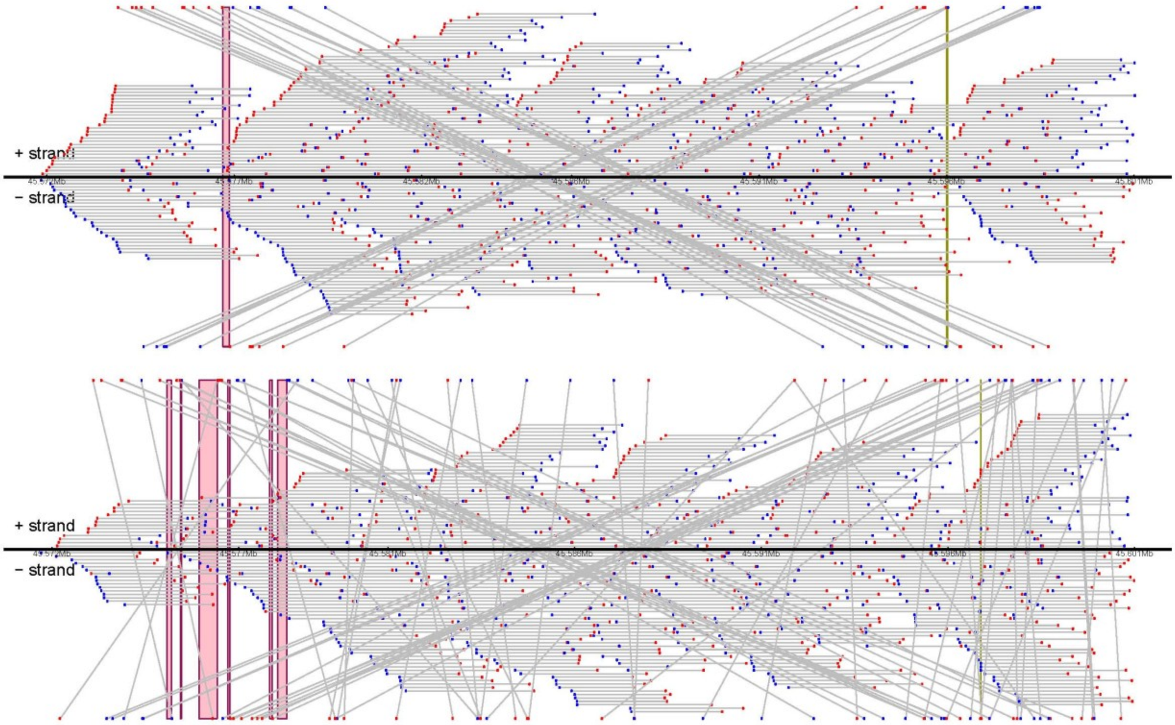

inversion (chr2:138720732−138726216)

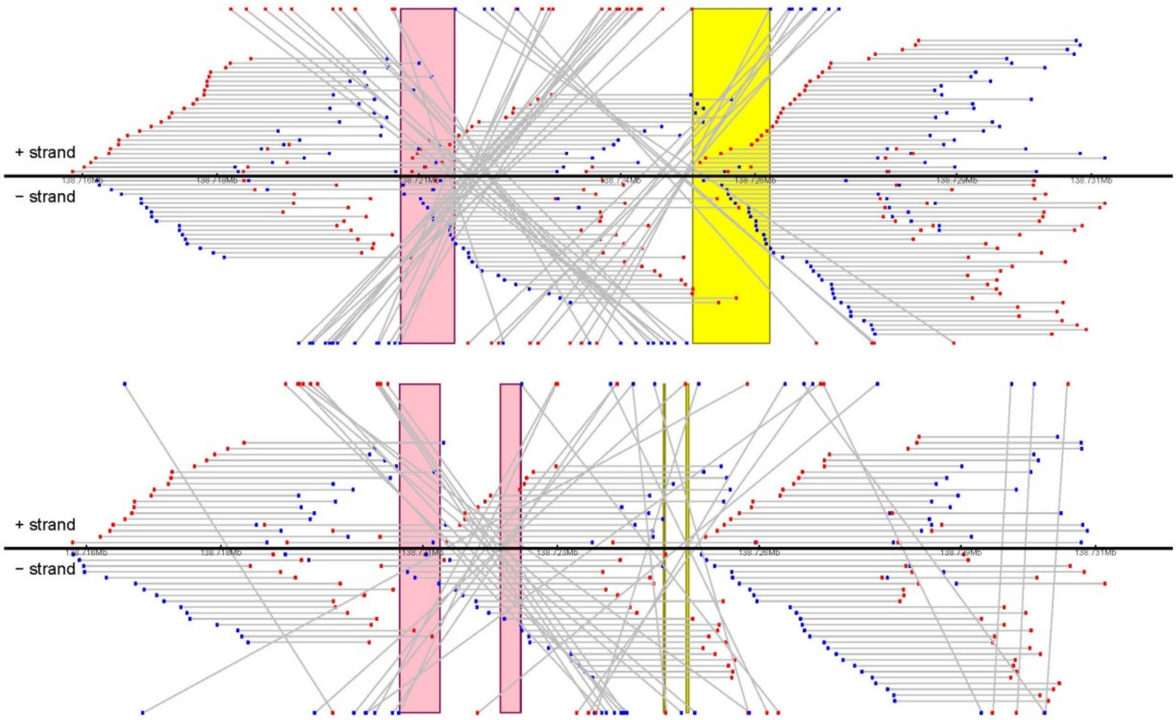

inversion (chr6:168834235−168837472)

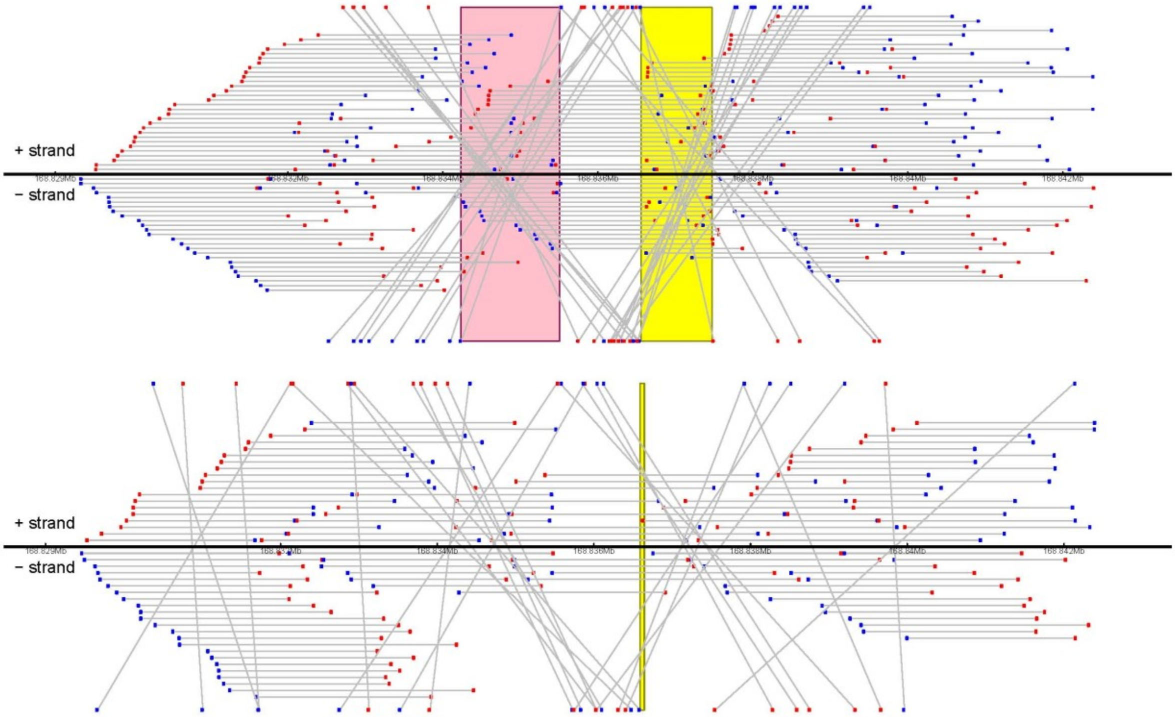

inversion (chr7:70062194−70077008)

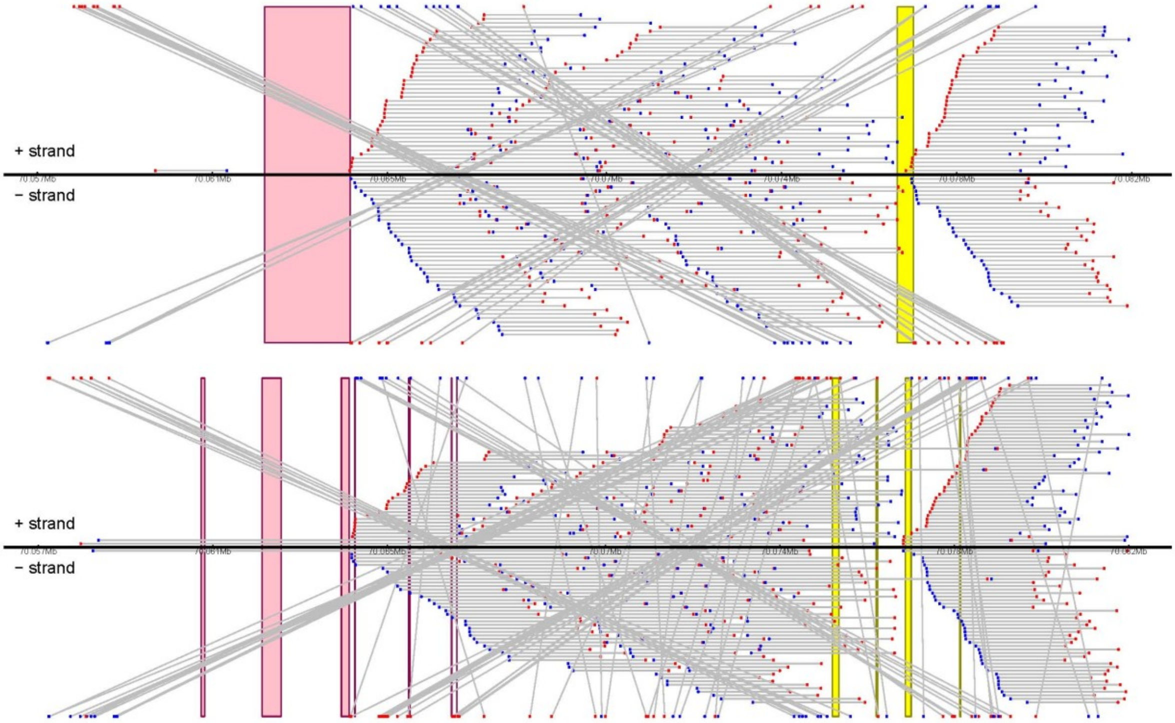

inversion (chr8:6141607−6145600)

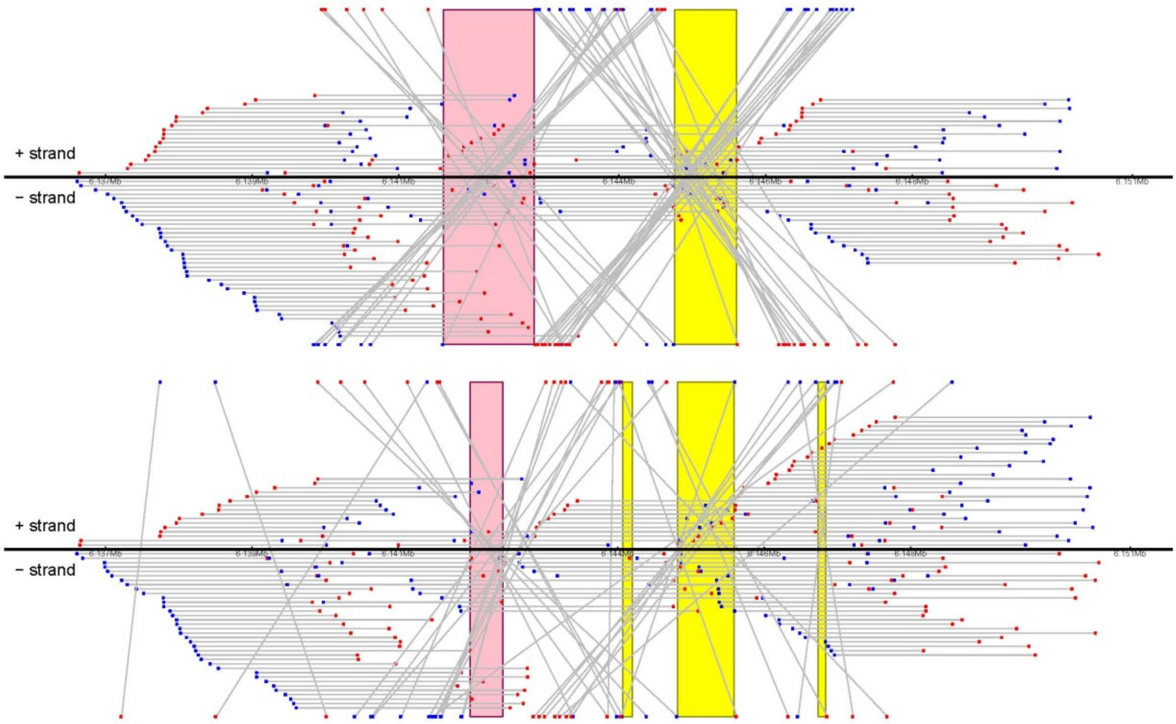

inversion (chr12:12435479−12438631)

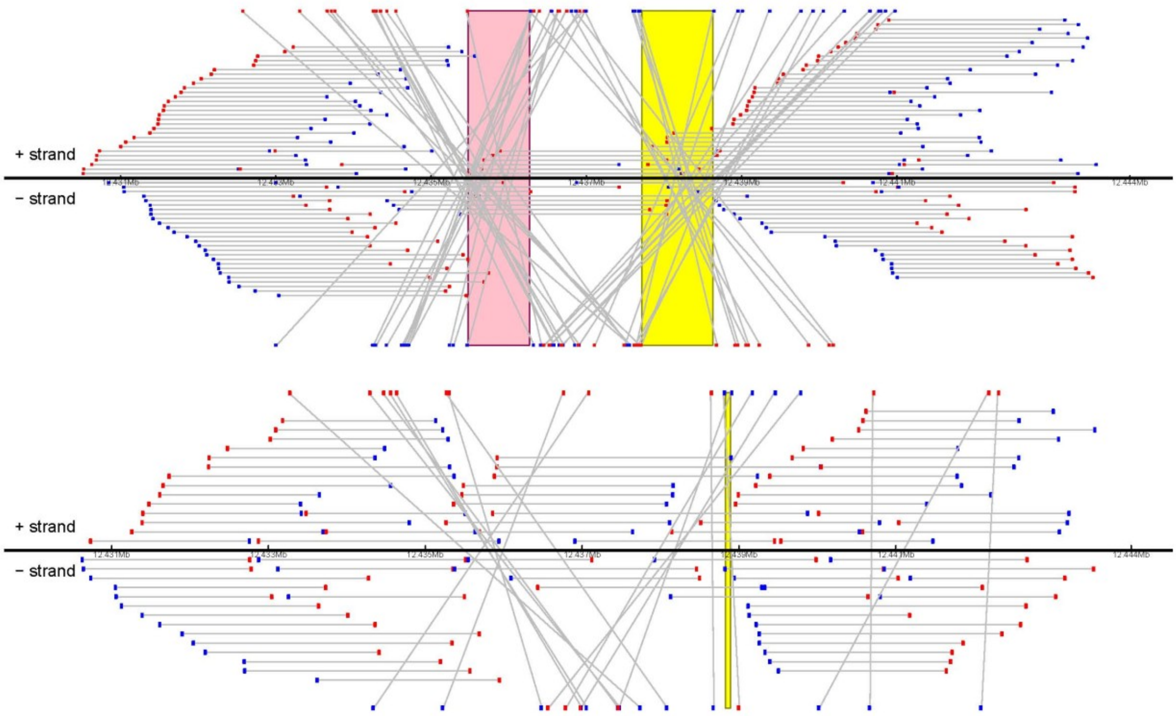

inversion (chr23:48899813−48907160)

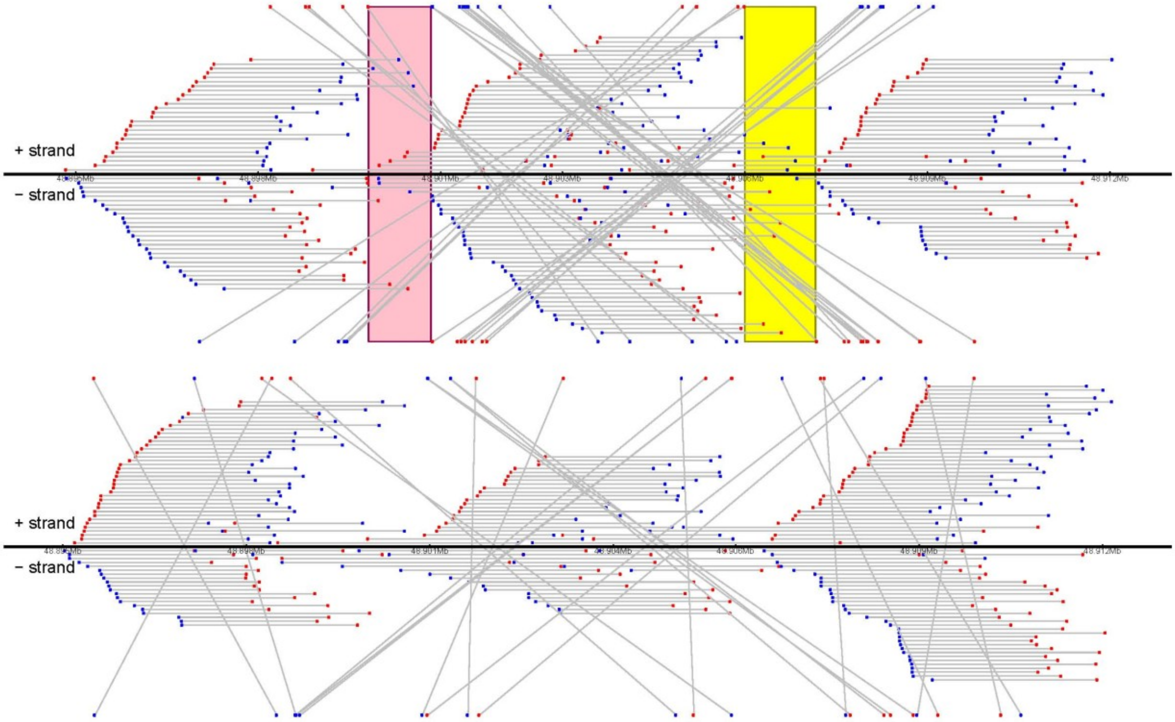

inversion (chr21:26294301−26298109)

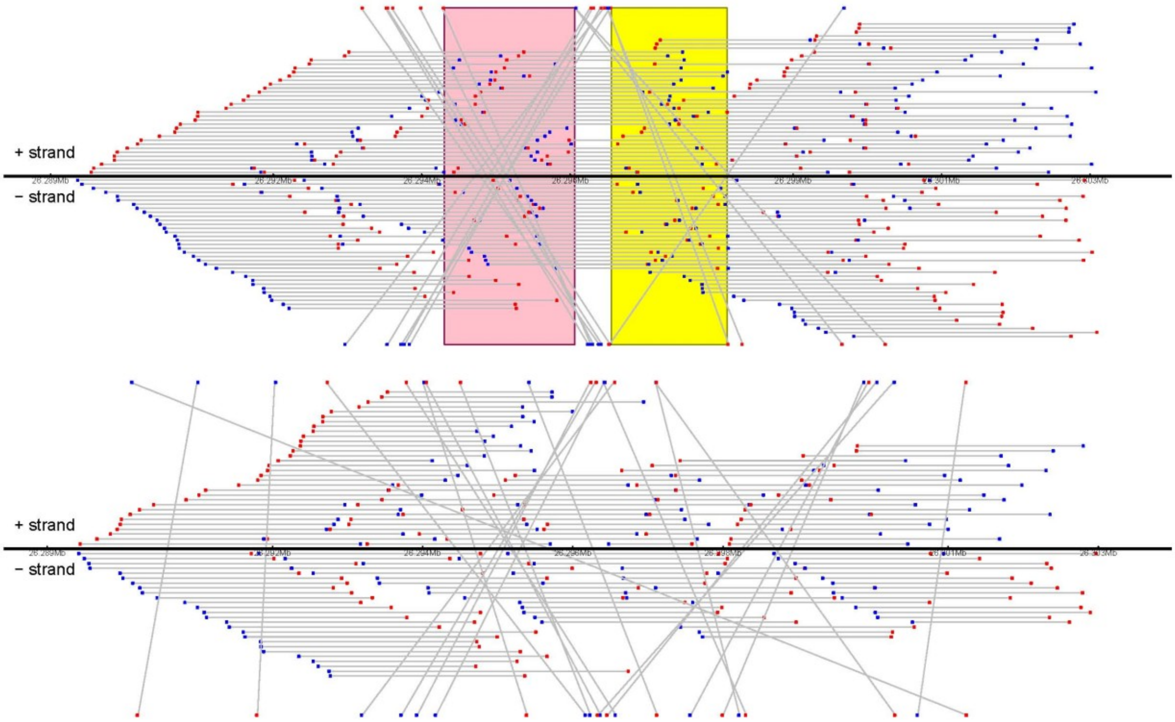

inversion (chr6:107274077−107278762)

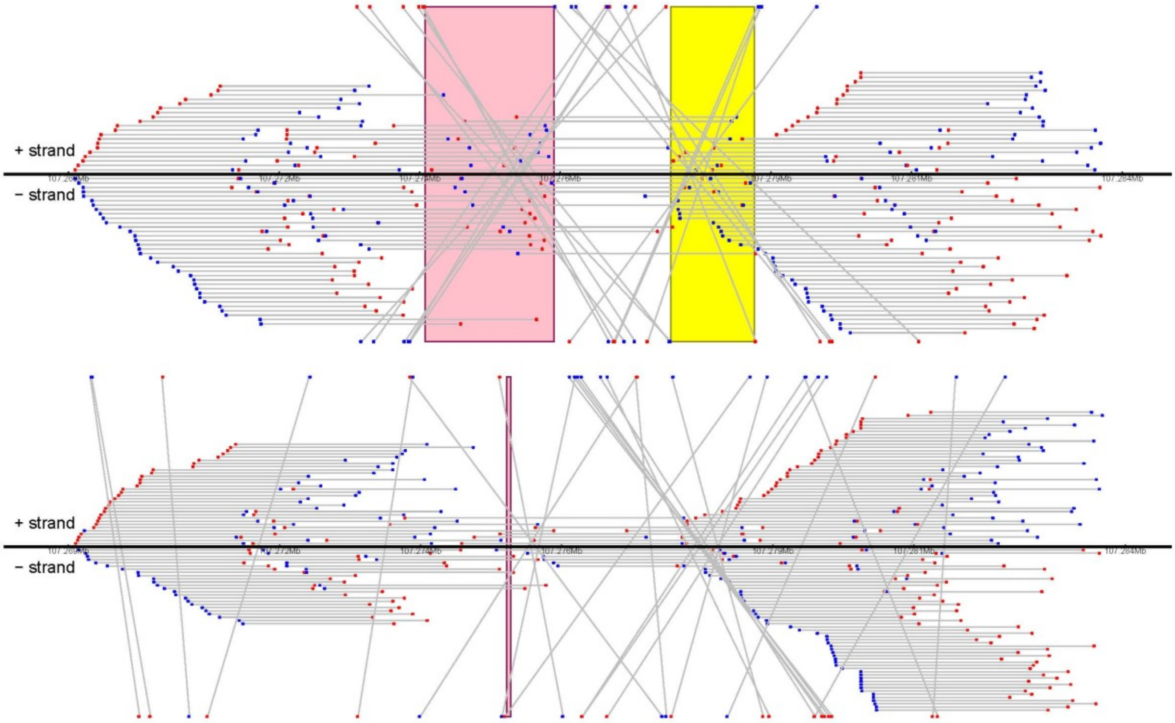

inversion (chr8:20820410−20823675)

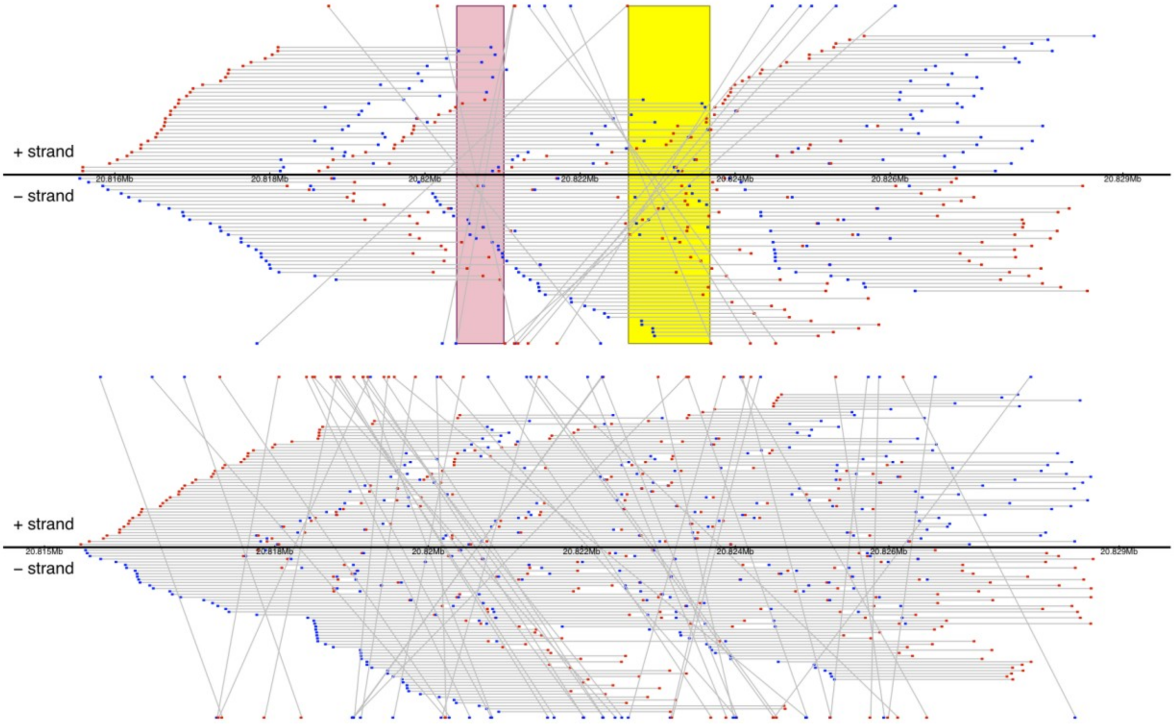

Supplement: Figure S4 — Loss of inversion detection sensitivity by spurious MDA-induced inversions. Ten examples of mate-paired data from true positive inversions in normal (upper panels) or MDA (lower panels) DNA from a healthy individual. Forward (blue) and reverse (red) tags in mate-pairs (grey line) are surrounding the start and end breakpoints along with 5 kb flanking regions on the chromosome (black line). The tags mapping to the forward chromosome strand are plotted above the chromosome line, and the tags mapping to the reverse strand are plotted below. The inversion start and end regions identified are shown as pink and yellow bands, respectively. (PDF) [file pone.0022250.s004.pdf]
