# Supplementary material for: Structural Alterations from Multiple Displacement Amplification of a Human Genome Revealed by Mate-Pair Sequencing
Source: PLoS One. 2011 Jul 22;6(7):e22250. doi: 10.1371/journal.pone.0022250 (PMC3142133; doi:10.1371/journal.pone.0022250)
Supplement: Table S1 — Primers used for PCR validation and sequencing. (PDF) [file pone.0022250.s006.pdf]

Table S1. Primers used for PCR validation and sequencing

Two primer pairs were designed for validation of each potential inversion (one pair at each breakpoint). Inversions were sequenced with their PCR primers. Three primer pairs were designed for each translocation. Universal sequencing primer 5'-GTAAAACGACGGCCAGT-3' was used for sequencing potential translocations.

Rearrangement type	Genomic position	Forward primer sequence	Reverse primer sequence
**Inversion**	chr2:138720732-138726216	TCCTGGTTTCTGATGTGAAGAA	AATCCAGAAAGAGACCCAGGTA
		CTGTATCAGTCACCACCCTGA	CACACACTCGCATTTTCTCTG
	chr4:190822785-190850470	GGGTATAAAGAAGGGGTTGGA	TGTCTTTCCTTTTTCCCAGGT
		TCCAATCCACAGGACAACAA	TGCTCCTTCCTCTTGTCTGG
	chr6:31117162-31119126	CCAGATTGCCACCTTTGAAC	TGGCATTCTGTGAAGGAACA
		CACTGTGTATAGAATGGGAAGCA	AGAGTAAATAGGTCCATCTTCTGG
	chr6:107274077-107278762	CCTCTTGCCACTCTCCACTA	GCATGGACAGCATTTGAAAG
		CAGAGCTGCTAGGACCAACC	AATCCACTCTTCCCCACCTC
	chr6:168834235-168837472	GTTTAGGTGCAGGGGACTGA	CCAAGCTAAAACGAGGTCACA
		GGTCTTCTGCACCTGGGATA	AGAAAAGGCCCACACAGGTA
	chr7:70062194-70077008	TGTTCAGTGTTCTTCATAATCTTCAA	AATCAACTGCTAACCCCAGAA
		TGATTGCTATTTCTGCCCATA	CACTGGGTCTTTGAGAAAAGG
	chr7:106845690-106850964	TTGAGGCCATTCTCAGAACA	CCAACGAAGAGGAAAAGGAG
		GCGTTTCTGTGCAAATACCA	TCTGGGCCTGTTGATTTCTT
	chr8:6141607-6145600	GGGCAGCAGAGCAAGACTAT	GCCCAGGCTATGTCACTGTT
		TCGGAATGATAACAGCTAAGTGA	GTTTATCAGCGCTTGGAAGG
	chr8:20820410-20823675	CCTCATGTGATTGTGTCTCGTT	TACCCCAGCATGGATTTCAC
		TATAAGGGCTTCCCCCTTTG	AGAAACCCTGCAACATTTGG
	chr10:46443056-46479589	GGGAACTGTGAGTGGTGGTG	TCGCATACAATAACACACTGC
		GCTCAGGATCCTTCAGGTTG	TGTGAGTGGCATGTGTTTCA
	chr10:127587152-127597234	GGAATGGAAAACCGAATATCA	GAAAAGTCAATTTCTTCTGTGTCA
		GGATGACTGCAGGCCTAATC	TCCTCATCTGTGAACCAGGA
	chr11:99194697-99196460	TCCAAGATCAGCAAATTGTCC	AAAGACTGCTCTACAGCTATTGG
		TGAGGTGTGTTCAATGTAGCA	TGTCGATGGATCCAAAAATG
	chr12:45576691-45596023	CAGCCCAACAAGAAAAATATTAAA	TGAAACATCTGCACAAGTATTGG
		GCAGGCACCAAGTTTCTGTT	TTCTCACATCAAGCCCTGAA
	chr12:12435479-12438631	GCTTGGTTCCTGCCATACTT	GTGCCTGGGACTACTGCTTC
		TGGACTTAGAGCTGCACCATT	CCAAGAAACAGGGAAAACCA
	chr16:83744887-83748786	GGCAGGGAGCTCATTCAATA	CTGCAGGTAAGGGACAGAGC
		CTTTGCTGGTGTTTTGCTCA	TTCTTTCTGGGCACTGTGTG
	chr21:26294301-26298109	ACATCCGCCGTAAAAGAATG	GGGCTTAGTGCTGACTGTCC
		TCACGTGGTTTTCAGATGGA	TCCCAGCTTCTCCCAGAGTA
	chrX:6145604-6149479	ATGGGAAGCCATGTGTGATT	ACAGGCACCTGTGAGCTTTT
		TGTGGTTTCTGTGCACCATT	TTTCTCAGCCATTGCAACAC
	chrX:26709863-26733708	GGAAGATTTTTCATTTGCCAGT	TGAATACAGGAAATCTTCATCAGA
		TTTTGAGTTTTGGGGGTATCA	GTCTTCTTCTCCCCCACCA
	chrX:46695829-46716139	GGCAGTCGCCTGTAATCC	CCAAATGGAAACTCTAGAACTGAA
		AAGAGGTCTTATCTGCAAATGG	AATGGTGCTAGCCCTTTCTAA
	chrX:48899813-48907160	ACTGCGATAACCCTCAAGGA	CATCCCAGAGTGGAAAAAGG
		CACCGGCGGTATAGGAGTT	GAATAACCCCAAGGCTCCTC
**Translocation**	chr1:154453166 - chr9:-6584750	GTAAAACGACGGCCAGTCTGGAGAGGTTGGGACTCTG	GCCTCAAAAGCTAATGAATGCT
		GTAAAACGACGGCCAGTCTGGAGAGGTTGGGACTCTG	AATCTGTGGCAAAGGACTGG
		GTAAAACGACGGCCAGTCTGGAGAGGTTGGGACTCTG	CAGAGCAAGACTCCATTAAGCA
	chr1:102627405 - chr21:-36167603	GTAAAACGACGGCCAGTTTTAAGGGCGGGAATTATCC	TGTTGAAAATAGATTATAATGGGAGA
		GTAAAACGACGGCCAGTTTTAAGGGCGGGAATTATCC	CCTCCAAGCCAACATAGAGC
		GTAAAACGACGGCCAGTTTTAAGGGCGGGAATTATCC	CTGCTTGGGCGACAGAGT
	chr2:3909520 - chr12:-123065704	GTAAAACGACGGCCAGTACCAAAAATTGTGCATGCTG	GTTTGGCATGGTGGATTTCT
		GTAAAACGACGGCCAGTACCAAAAATTGTGCATGCTG	GATGGGAGCAACCCAAACTA
		GTAAAACGACGGCCAGTACCAAAAATTGTGCATGCTG	TTGGGCAAATCGCTTAATCT
	chr2:3912660 - chr12:-123065054	GTAAAACGACGGCCAGTGCCACTTGGTGTCAAGTCAA	ATGGGTGTGTGGTGGAGAAT
		GTAAAACGACGGCCAGTGCCACTTGGTGTCAAGTCAA	ATGCCGAGGTGGTCAGTATC
		GTAAAACGACGGCCAGTGCCACTTGGTGTCAAGTCAA	TGCATTGGAAAACAGACAGC
	chr2:227905478 - chr15:-91639828	GTAAAACGACGGCCAGTCCTCCCGCTTTTCTCTTCTT	AGCTCTGGCCTAAGGAAACC
		GTAAAACGACGGCCAGTCCTCCCGCTTTTCTCTTCTT	AGAAGAAACAGCTCACCTCTAAAT
		GTAAAACGACGGCCAGTCCTCCCGCTTTTCTCTTCTT	TCTTGGCATCCCAGGAAA

Rearrangement type	Genomic position	Forward primer sequence	Reverse primer sequence
	chr2:227930736 - chr15:-91638688	GTAAAACGACGGCCAGTCCAGAAAGTCTGGAGGAAGG	AGATGATCTCCCACCCTGTG
		GTAAAACGACGGCCAGTCCAGAAAGTCTGGAGGAAGG	GGGATGAGTACGGAGAGGTG
		GTAAAACGACGGCCAGTCCAGAAAGTCTGGAGGAAGG	CCAACATGCCTGGCTAAT
	chr2:229753826 - chr17:30502256	GTAAAACGACGGCCAGTTTCGTATGCCATCAACTCCA	TATGCTCCTGGCTCACATCA
		GTAAAACGACGGCCAGTTTCGTATGCCATCAACTCCA	CCCTGGGTCCTCCATAAAGT
		GTAAAACGACGGCCAGTTTCGTATGCCATCAACTCCA	CAAAATTTAAAGGGGCACCA
	chr3:37724462 - chr17:-10600599	GTAAAACGACGGCCAGTGAAAGTGGTGTGTGGACAGG	TGTGACCCTGACCAATGAAA
		GTAAAACGACGGCCAGTGAAAGTGGTGTGTGGACAGG	AAGGAACCAAGCGGGTAGAT
		GTAAAACGACGGCCAGTGAAAGTGGTGTGTGGACAGG	TATTCAGTGCAGGGGAGGAG
	chr4:49211689 - chr2:161843571	GTAAAACGACGGCCAGTTTCAGCGGGAGTCCATTG	GGAAGGAGAAAACACCCAGTG
		GTAAAACGACGGCCAGTTTCAGCGGGAGTCCATTG	GCACCTGTCAACCCAACAC
		GTAAAACGACGGCCAGTTTCAGCGGGAGTCCATTG	CCTGATGCATGGCAGTTTAAG
	chr4:77026243 - chr19:-23975976	GTAAAACGACGGCCAGTGCGAGAAGAGCGAAACTCTG	ACCAGCTTGAGGTTGCATTC
		GTAAAACGACGGCCAGTGCGAGAAGAGCGAAACTCTG	AACTGGAGAGGCCTCAGGAT
		GTAAAACGACGGCCAGTGCGAGAAGAGCGAAACTCTG	GCCCTGCTCTTCACAAAAAG
	chr7:57553423 - chr10:-39146855	GTAAAACGACGGCCAGTATTGTGATCGTACGGGAAGG	TTTCTTTTGACTCCGTTCCA
		GTAAAACGACGGCCAGTATTGTGATCGTACGGGAAGG	GCCATTCCATTCGGCTCTA
		GTAAAACGACGGCCAGTATTGTGATCGTACGGGAAGG	CCAAACAATTTGATGCCATT
	chr8:47023896 - chr9:66563319	GTAAAACGACGGCCAGTATGTCCATTCAACAAACAGAGA	TCACTTTATCAACGAAATCCTCA
		GTAAAACGACGGCCAGTTGTTGCCTATGGTGGAATTG	GCGCTGCAAATCTCTACTTT
		GTAAAACGACGGCCAGTTGTTGCCTATGGTGGAATTG	AACGAAGGCCTCAAAGCTG
	chr8:47023898 - chr11:-50394476	GTAAAACGACGGCCAGTTTGGAGCGCTTTGAGACC	CATTCGCAGAATGAACAGAA
		GTAAAACGACGGCCAGTTTGGAGCGCTTTGAGACC	TCCACACCGGTGAATCAAAT
		GTAAAACGACGGCCAGTTTGGAGCGCTTTGAGACC	GCAAATCAGTCCCAGAAAGC
	chr9:66565153 - chr8:47023892	GTAAAACGACGGCCAGTTTTCATTGGAAGCGGGAATA	TCAGCTGTAGACCTCAAAGCA
		GTAAAACGACGGCCAGTTTTCATTGGAAGCGGGAATA	TTCAGTGAATTGCTTCGGTCT
		GTAAAACGACGGCCAGTTTTCATTGGAAGCGGGAATA	TTGGCCTCAAAGCACTCC
	chr12:55869932 - chr18:35575494	GTAAAACGACGGCCAGTTAAGGATCGCCTAGGAGCAA	CTGGTTACATGCCTGGCTTT
		GTAAAACGACGGCCAGTTAAGGATCGCCTAGGAGCAA	TTGGTCTGCGTCTACTGCAC
		GTAAAACGACGGCCAGTTAAGGATCGCCTAGGAGCAA	ATTAGGGTGGTGTCCAGCAG
	chr12:34263931 - chr19:23975948	GTAAAACGACGGCCAGTCTACGATGAGGGGCTCTCTG	GTGGGCATGGAAGTCAGAAT
		GTAAAACGACGGCCAGTCTACGATGAGGGGCTCTCTG	AATCCCGGGACTGAGGTAGT
		GTAAAACGACGGCCAGTCTACGATGAGGGGCTCTCTG	CTGGACGGTGAGGGTACTGT
	chr16:75421396 - chr7:127717694	GTAAAACGACGGCCAGTGCTCTTGCATAGCATTGGTG	TGCATGTAATCCTGAGCTCTT
		GTAAAACGACGGCCAGTGCTCTTGCATAGCATTGGTG	CTGACCCCACAGGCAGTC
		GTAAAACGACGGCCAGTGCTCTTGCATAGCATTGGTG	CCAGCTGAGTGCTGGAAGA
	chr16:34520039 - chr18:-99338	GTAAAACGACGGCCAGTAACCAGGGAGGAAAGTCACA	TCTCCAATGCTCACTCAGGA
		GTAAAACGACGGCCAGTAACCAGGGAGGAAAGTCACA	CCCCACCTACAGGGGGTAT
		GTAAAACGACGGCCAGTAACCAGGGAGGAAAGTCACA	TGCCTACAGGGAATTTCGAC
